# Progression of precancerous lesions of esophageal squamous cell carcinomas in a high‐risk, rural Chinese population

**DOI:** 10.1002/cam4.4965

**Published:** 2022-06-30

**Authors:** Dongqing Gao, Peipei Lu, Nan Zhang, Li Zhao, Jinhui Liu, Jia Yang, Jingmin Liu, Deli Zhao, Jialin Wang

**Affiliations:** ^1^ Jining NO.1 People's Hospital Jining China; ^2^ Shandong Cancer Hospital and Institute Shandong First Medical University and Shandong Academy of Medical Sciences Jinan China; ^3^ Cancer Center, People's Hospital of Feicheng Taian China; ^4^ School of Public Health Weifang Medical University Weifang China

**Keywords:** annual transition probability, esophageal squamous cell carcinoma, follow‐up, natural history, precancerous lesions

## Abstract

**Background and aims:**

This study investigated the natural history of esophageal squamous cell carcinoma (ESCC) in rural Chinese. We sought to help provide more data to support ESCC screenings.

**Methods:**

This study was based on an existing Screening Program in Feicheng, China. Esophageal precancerous lesions were identified in 1753 cases, diagnosed from esophageal cancer screenings from 2006 to 2016. We followed up with them through endoscopic screening until October 1, 2017. Pathology results from various grades of precancerous lesions were recorded and the annual transition probabilities and incidence density of ESCC were calculated.

**Results:**

As of October 1, 2017, a total of 4055.8 person‐years has been observed. The ESCC incidence density of mild, moderate, and severe dysplasia (SD) was 0.17, 0.79, and 1.77 per 100 person‐years, respectively. The median follow‐up time of mild, moderate, and SD was 3.5, 2.3, and 2.2 years, respectively. The annual transition probability of mild, moderate, and SD to the next pathological level was 0.025, 0.038, and 0.016, respectively. The ESCC incidence density of males was 2.6 times higher than females (0.58 vs. 0.22), and the older age group (56–69 age group) had a ESCC incidence density 1.2 times higher than the younger group (40–55 age group) (0.45 vs. 0.39).

**Conclusions:**

The higher the grade of precancerous lesions, the higher the incidence density of ESCC. Screening of esophageal cancer in males and the elderly should be strengthened. It is recommended to reinforce follow‐up management for untreated patients with SD/carcinoma in situ. For patients with mild and moderate dysplasia in high‐risk rural Chinese populations, endoscopic follow‐up intervals can be appropriately adjusted to once every 2 years.

## Graphical abstract

This study aimed to investigate the natural history of esophageal squamous cell carcinoma (ESCC) and estimate the annual transition probability of precancerous lesions of ESCC in a rural Chinese population.The ESCC incidence density of mild, moderate and severe dysplasia was 0.17, 0.79 and 1.77 per 100 person‐years, respectively.The annual transition probability of mild, moderate and severe dysplasia to next pathological level was 0.025, 0.038 and 0.016, respectively.

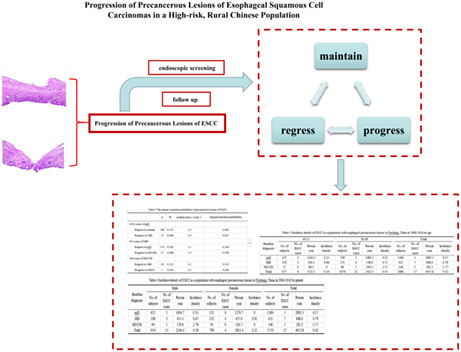

## 1 INTRODUCTION

Esophageal cancer is one of the most common types of malignant tumors in the world. Globally, it is the seventh most common cancer and the sixth most common cause of cancer death, with estimations of 572,000 new cases and 509,000 deaths.^1^ Approximately 54% of esophageal cancer cases worldwide occur in China.^2^ Esophageal squamous cell carcinoma (ESCC) is the sixth most common cancer and the fourth most common cause of cancer death in China, with estimations of 246,000 new cases and 188,000 deaths in 2015,^3^ is the main pathological type in China.^4^ Since esophageal cancer has no specific symptoms in its early stage, more than 90% of the patients are diagnosed at middle or advanced stages. It has a poor prognosis with the 5‐year age‐standardized relative survival rate of only 20.9%.^5^ Even though the mortality of esophageal cancer has noticeably dropped in the past three decades in China, it is still the main cancer burden, especially in rural areas.^6^ Effective means to reduce the incidence and mortality of ESCC include secondary prevention by early screening and tertiary prevention by early intervention of esophageal precancerous lesions.

The occurrence and development of ESCC are a multi‐factorial, multi‐stage, and long‐term process, which develops through basal cell hyperplasia (BCH), esophagitis, mild dysplasia (mD), moderate dysplasia (MD), and severe dysplasia/carcinoma in situ (SD/CIS).^7^, ^8^ Squamous dysplasias have been proven to be significant precursor lesions of esophageal cancer; increasing grades of dysplasia were strongly associated with increased risk.^9^, ^10^ Exploration of population distribution and natural outcome characteristics of patients with esophageal precancerous lesions via endoscopic screening with long‐term follow up care provides critical information for ongoing research on prevention and treatment of ESCC. The National Cancer Early Detection and Treatment Project (NCEDTP) on upper gastrointestinal cancer in rural areas has already been carried out for more than 10 years, covering 29 provinces, autonomous regions, and municipalities directly under the Central Government. A total of 2 million people have participated in endoscopic screenings, and 32,000 patients have been identified with a detection rate of 1.69%, an early diagnosis rate of 72.64%, and a treatment rate of 83.38%.^11^ The cumulative mortality rate of ESCC has been reduced by 33.7% over 10 years.^12^

Feicheng city, Shandong, began to implement the Esophageal Cancer Screening Program in 2006. However, there are few studies on long‐term surveillance of different grades of ESCC in China.^13^ In addition, the current NCEDTP screening protocol was based on evidence obtained from 682 patients with cytological abnormalities from an extremely high‐risk area for ESCC in 2005.^14^ It is necessary to optimize the screening guidelines of ESCC with the increasing implementation of the NCEDTP screening program.

Our study aims to investigate the natural history of ESCC in a rural Chinese population.

## 2 MATERIAL AND METHODS

### 2.1 Study population

Feicheng city, Shandong began to undertake the NCEDTP^11^, ^15^ for upper gastrointestinal cancer in 2006. This study was based on the existing Esophageal Cancer Screening Program, and the objective was to investigate the characterize the outcomes of ESCC precancerous lesions. The study was approved by the Institutional Ethical Review Board of Shandong Cancer Hospital and Institute, which waived individual consent for the use of registry data.

**FIGURE 1:**
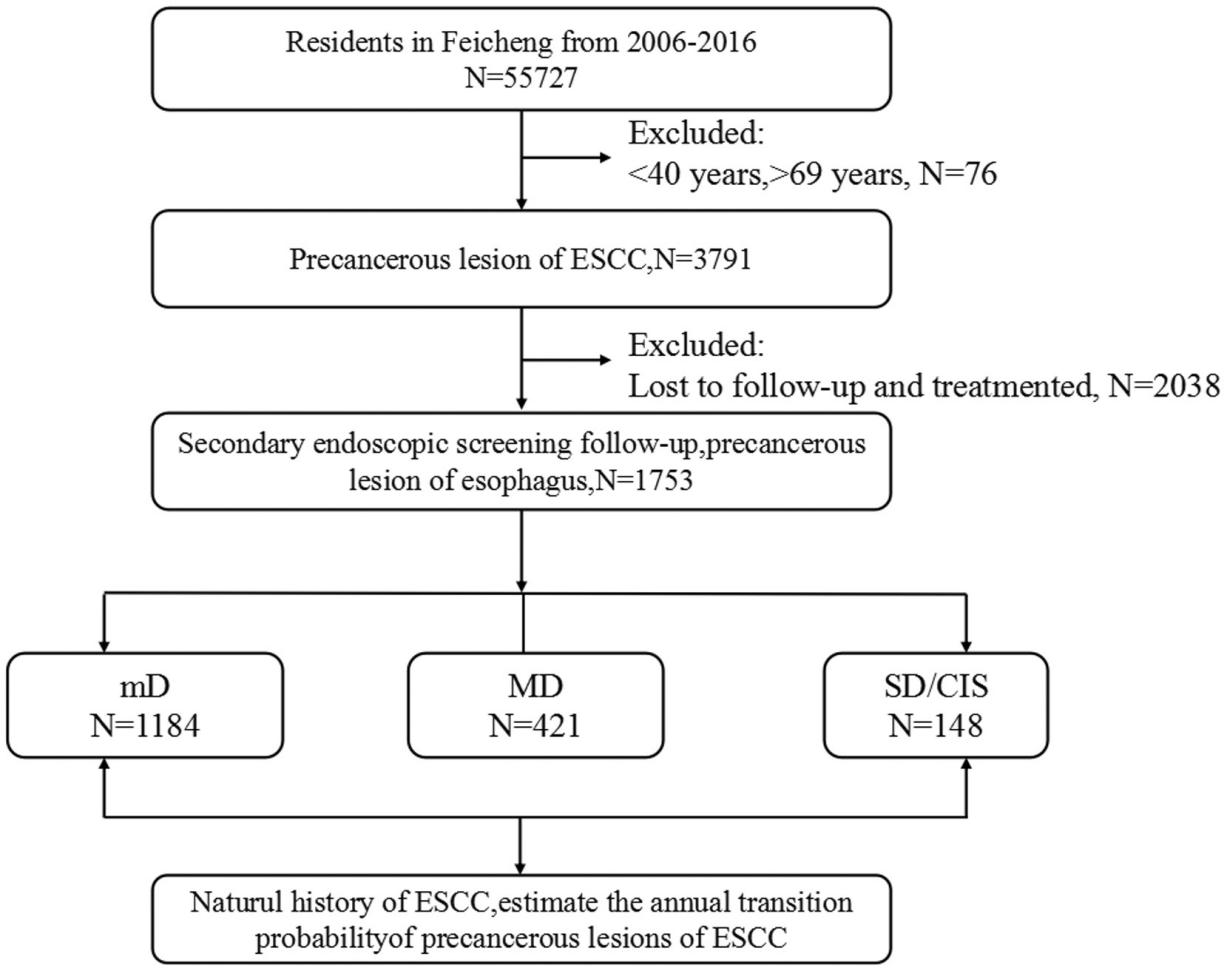
Flowchart of participants included in the study. mD, mild dysplasia; MD, moderate dysplasia; SD/CIS, severe dysplasia/carcinoma in situ.

Rural residents aged 40 to 69 years were screened using “iodine staining observation under endoscope + indicative biopsy” if they met the following inclusion criteria: (1) 40–69 years old; (2) local residents; (3) voluntarily signed informed consent; (4) healthy, no patients with significant organ diseases, such as heart, brain, lung, liver, and kidney disease. The screening contraindications were: (1) Patients with severe heart disease or heart failure; (2) patients with severe respiratory diseases, dyspnea, and/or asthma; (3) severe digestive system diseases, mental disorders, coagulation disorders, etc.; (4) pregnancy; (5) contraindications to endoscopic examination; (6) have a history of iodine allergy. The study design and flowchart are shown in Figure 1.

### 2.2 Screening procedures

The residents who met the inclusion criteria participated in both a group education and an individual conversation, and the informed consent form was signed on a voluntary basis. Before the endoscopic screening examination, the basic information questionnaire was conducted by well‐trained interviewers. The questionnaire included questions on demographics (age, gender, family status education level, etc.), living habits (cigarette use, alcohol consumption, drinking tea, etc.), medical history of digestive system diseases, family history of any type of cancer, etc.

The screening procedures were in accordance with clinical guidelines and were consistent with those used in previous studies.^6^, ^7^ The subject was next placed in a left decubitus position and the endoscope was inserted through the mouth. At 16 cm from the incisors, the endoscope was slowly advanced and the status of esophageal mucosa was carefully observed for every 1 cm. After the endoscopic examination, withdraw the endoscope at about 20 cm of the incisor, and send the iodine solution from the endoscopic biopsy tube into the nozzle. While the endoscope is slowly advanced, 20 ml of 1.2%–1.5% iodine solution is taken from the nozzle. Inject, the iodine solution is evenly sprayed on the esophageal mucosa from top to bottom, and finally, the staining of the esophageal mucosa was carefully observed. Carefully observe the depth of yellow in the unstained area with a clear boundary. According to the degree of dysplasia of the lesion, the color of the unstained area varies from light yellow to dark yellow. Medical staff should record in detail the size, distance, state, and clock orientation of the mucosa in the yellow area from the incisor, and perform multiplebiopsies of the mucosa in the yellow area based on the size of the lesion.

The biopsy slides were read jointly by two experienced pathologists who did not know the visual endoscopic findings. If there is a disagreement, the biopsy specimen will be sent to the National Health Commission for evaluation by the expert committee. The pathological diagnosis results were normal, BCH, esophagitis, mD, MD, SD/CIS, and ESCC.^7^, ^16^ The diagnostic criteria of pathology is according to National Cancer Center/National Clinical Research Center for Cancer, Department of Cancer Registry of China.^19^ According to the screening protocol, all subjects diagnosed with SD/CIS and ESCC should be treated with endoscopy or surgery in principle.

### 2.3 Follow‐up procedures

The subjects with mD were followed up with every 3 years, those with MD were followed up with once a year, those with SD/CIS should complete clinical treatment, and those who refused treatment were followed up with at least once a year. The follow‐up end date was October 1, 2017 in this study. Endoscopic iodine staining was also used in the follow‐up, and indicative biopsy and pathological diagnosis were required for positive iodine staining. All follow‐up data were collected by one‐on‐one project staff. Based on the follow‐up pathological results, reduction or disappearance of the lesion was defined as regression, the grade of the lesion not changing as stable, and the worsening of the lesion as progression. The incidence density of ESCC was defined as the number of ESCC cases/total number of follow‐up person years.

### 2.4 Quality control

All medical staff involved in the project were trained for the required qualifications and working background. Quality control personnel were placed in every aspect of on‐site work to ensure the quality of work. The endoscopists participating in the screening have been well‐trained by experts from the Cancer Institute of the Chinese Academy of Medical Sciences and have extensive clinical experience. To improve the management of the follow‐up subjects, special personnel were organized to notify the subjects of regular follow‐up endoscopic examinations. The follow‐up procedure was carried out strictly in accordance with the follow‐up principles formulated by the technical plan.

### 2.5 Statistical analyses

Chi‐squared tests or Fisher exact tests were used to compare the basic demographic characteristics among different groups, and Cochran‐Armitag method was used for the trend test. The incidence density of ESCC in different gender and age groups as of October 1, 2017 was calculated in person‐years. Chi‐squared tests or Fisher exact tests were used to compare the difference in the incidence density of ESCC in precancerous lesions between genders, ages, and pathological grades. The transition probabilities were calculated by an equation:

$$p=1–e^{-\mathrm{rt}},$$

$$r=-\ln\left(1-P_{0}\right)/t_{0},$$

*P*_0_ means the proportion of transition from one state to another at *t*_0_ time. *p* is the annual transition probability when *t* is 1 year.^17^ All analyses were performed using SPSS22.0, all tests were two‐sided, and statistical significance was accepted as *p* ≤ 0.05 in this study.

## 3 RESULTS

### 3.1 Baseline situation

From 2006 to 2016, a total of 1753 individuals were included in our analysis, including 1184 cases of mD, 421 cases of MD, and 148 cases of SD/CIS, who were refused treatment. In the study, there were 954 males and 799 females. Table 1 shows the basic demographic characteristics based on pathological diagnosis at baseline. There were statistically significant differences in gender, age, BMI in our study population(*p* ≤ 0.05).

**TABLE 1:** The basic demographic characteristics of esophageal squamous cell carcinoma screening population in Feicheng, China in 2006–2016

Variable	Total *n* (%)	mD *n* (%)	MD *n* (%)	SD/CIS *n* (%)	*p* value for trend
Gender					0.664
Male	954 (54.4)	652 (55.1)	208 (49.4)	94 (63.5)	
Female	799 (46.6)	532 (44.9)	213 (50.6)	54 (36.5)	
Age (years)					0.021
40–49	310 (17.7)	235 (19.8)	57 (13.5)	18 (12.2)	
50–59	716 (40.8)	473 (39.9)	177 (42.0)	66 (44.6)	
60–69	727 (41.5)	476 (40.2)	187 (44.4)	64 (43.2)	
Education					0.159
Primary school and below	927 (52.9)	610 (51.5)	236 (56.1)	81 (54.7)	
Middle school and above	826 (47.1)	574 (48.5)	185 (43.9)	67 (45.3)	
Smoking					0.771
No	1194 (68.1)	797 (67.3)	302 (71.7)	95 (64.2)	
Yes	559 (31.9)	387 (32.7)	119 (28.3)	53 (35.8)	
Body mass index					0.350
<18.5	48 (2.7)	27 (2.3)	16 (3.8)	5 (3.4)	
18.5–23.9	1129 (64.4)	748 (63.2)	272 (64.6)	109 (73.6)	
≥24.0	524 (29.9)	409 (34.5)	133 (31.6)	33 (23.0)	
History of digestive system disease					0.537
No	1484 (84.7)	1009 (85.2)	349 (82.9)	126 (85.1)	
Yes	269 (15.3)	175 (14.8)	72 (17.1)	22 (14.9)	
Family history of tumor					0.023
No	1317 (75.1)	871 (73.6)	327 (77.7)	119 (80.4)	
Yes	436 (24.9)	313 (26.4)	94 (22.3)	29 (19.6)	
Total	1753	1184	421	148	

Abbreviations: CIS, carcinoma in situ; mD, mild dysplasia; MD, moderate dysplasia; SD, severe dysplasia.

### 3.2 Follow‐up situation

After a median follow up time of 2.3 (1.2–2.9) years, a total of 1090 cases (62.2%, 1090/1753) regressed, 520 (29.7%, 520/1753) maintained the original pathological grades, and 143(8.2%, 143/1753) progressed. The median time of regression, maintaining stability, and progression were 2.2, 2.4, and 2.3 years, respectively. During the follow‐up period, 232(13.2%, 232/1753) cases (160 mD, 51 MD, and 21 SD/CIS) improved to normal mucosa. 17(0.97%, 17/1753) newly diagnosed ESCC cases were developed from 5 mD, 7 MD, and 5 SD/CIS, respectively, and a median progress time of mD, MD, and SD/CIS were 3.5, 2.3, and 2.2 years, respectively. Among the 1184 mD cases at baseline, 18 (1.5%) cases progressed to SD/CIS, and 5 (0.4%) cases progressed to ESCC with a median time of 1.4 and 3.5 years, respectively. Of the 421 MD cases at baseline, 37 (8.8%) cases progressed to SD/CIS, and 7 (1.7%) cases progressed to ESCC with a median time of 2.3 and 2.3 years, respectively. Of the 148 SD/CIS cases at baseline, 5 (3.4%) cases progressed to ESCC with a median time of 2.2 years. For the high‐grade precancerous lesions, the median time to progression to ESCC was shorter than that of low‐grade precancerous lesions. Table 2 shows the specific results and median time of each grade of precancerous lesions during the follow‐up period.

**TABLE 2:** Pathological results of precancerous lesions of the esophagus after mean/median follow‐up of 2.2 years

Baseline diagnosis	No. of subjects in follow‐up	Normal		BCH		Esophagitis		mD		MD		SD/CIS		ESCC	
		*n* (%)	Median time	*n* (%)	Median time	*n* (%)	Median time	*n* (%)	Median time	*n* (%)	Median time	*n* (%)	Median time	*n* (%)	Median time
mD	1184	160 (13.5)	2.3	88 (7.4)	2.5	422 (35.6)	2.4	420 (35.5)	2.5	71 (6.0)	2.4	18 (1.5)	1.4	5 (0.4)	3.5
MD	421	51 (12.1)	1.3	23 (5.5)	1.4	67 (15.9)	1.4	153 (36.3)	1.4	83 (19.7)	1.8	37 (8.8)	1.5	7 (1.7)	2.3
SD/CIS	148	21 (14.2)	1.0	10 (6.8)	1.5	29 (19.6)	1.3	48 (32.4)	1.4	18 (12.2)	1.1	17 (11.5)	2.4	5 (3.4)	2.2

*Note*: Median time is in units of years. A total of 1090 cases (1090/1753, 62.2%) regressed, 520 (520/1753, 29.7%) were stable, and 143 (143/1753, 8.2%) progressed. The median time of regression, staying stable, and progression were 2.2, 2.4 and 2.3 years, respectively.

Abbreviations: CIS, carcinoma in situ; ESCC, esophageal squamous cell carcinoma; mD, mild dysplasia; MD, moderate dysplasia; SD, severe dysplasia.

The annual transition probabilities from mD to normal and MD were 0.061and 0.025, respectively. The annual transition probabilities from MD to mD and SD/CIS were 0.194 and 0.038, respectively. The annual transition probabilities from SD/CIS to MD and ESCC were 0.111 and 0.016, respectively **(**Table 3 **)**.

**TABLE 3:** The annual transition probability of precancerous lesion of ESCC

	*n*	*P*_0_	Median time (years)	Annual transition probability
1184 cases of mD				
Regress to normal	160	0.135	2.3	0.061
Progress to MD	71	0.060	2.4	0.025
421 cases of MD				
Regress to mD	153	0.363	2.1	0.194
Progress to SD/CIS	37	0.088	2.3	0.038
148 cases of SD/CIS				
Regress to MD	18	0.122	1.1	0.111
Progress to ESCC	5	0.034	2.2	0.016

Abbreviations: CIS, carcinoma in situ; ESCC, esophageal squamous cell carcinoma; mD, mild dysplasia; MD, moderate dysplasia; SD, severe dysplasia.

Tables 4 and 5 show the incidence density of ESCC among those with esophageal precancerous lesions in Feicheng, China in 2006 to 2016 by gender and age. As of October 1, 2017, a total of 4055.8 person‐years were observed. There were 17 new cases of ESCC diagnosed during the follow‐up period with an incidence density of 0.42 (17/4055.8) per 100 person‐years. The incidence densities of mD, MD and SD/CIS were 0.17, 0.79, and 1.77 per 100 person‐years, respectively, the higher grades of dysplasia having a higher ESCC incidence density (*p* < 0.05). The stratified analysis by gender and age group showed that the incidence density of ESCC in males is higher than that in females (0.58 vs. 0.22, respectively) (*p* > 0.05). The 56–69 age group had a higher incidence density of ESCC than the younger group (0.45 vs. 0.39, respectively) (*p* > 0.05).

**TABLE 4:** Incidence density of ESCC in a population with esophageal precancerous lesions in Feicheng, China in 2006–2016 by gender

Baseline diagnosis	Male				Female				Total			
	No. of subjects	No. of ESCC cases	Person year	Incidence density	No. of subjects	No. of ESCC cases	Person year	Incidence density	No. of subjects	No. of ESCC cases	Person year	Incidence density
mD	652	5	1614.7	0.31	532	0	1270.7	0	1184	5	2885.5	0.17
MD	208	3	451.1	0.67	213	4	437.0	0.92	421	7	888.0	0.79
SD/CIS	94	5	178.6	2.78	54	0	103.7	0	148	5	282.3	1.77
Total	954	13	2244.4	0.58	799	4	1811.4	0.22	1753	17	4055.8	0.42

*Note*: Comparing the incidence density of ESCC of mD, MD and SD/CIS group, the *p* value ≤0.00; the incidence density was compared by gender. *p* value = 0.067.

Abbreviations: CIS, carcinoma in situ; ESCC, esophageal squamous cell carcinoma; mD, mild dysplasia; MD, moderate dysplasia; SD, severe dysplasia.

**TABLE 5:** Incidence density of ESCC in a population with esophageal precancerous lesions in Feicheng, China in 2006–2016 by age

Baseline diagnosis	40–55				56–69				Total			
	No. of subjects	No. of ESCC cases	Person year	Incidence density	No. of subjects	No. of ESCC cases	Person year	Incidence density	No. of subjects	No. of ESCC cases	Person year	Incidence density
mD	475	3	1204.3	0.25	709	2	1681.1	0.12	1184	5	2885.5	0.17
MD	150	3	240.1	0.88	271	4	548.0	0.73	421	7	888.0	0.79
SD/CIS	52	0	88.1	0	96	5	194.2	2.57	148	5	282.3	1.77
Total	677	6	1532.5	0.39	1076	11	2423.3	0.45	1896	17	4055.8	0.42

*Note*: The incidence density of ESCC was compared by age group. *p* value = 0.777.

Abbreviations: CIS, carcinoma in situ; ESCC, esophageal squamous cell carcinoma; mD, mild dysplasia; MD, moderate dysplasia; SD, severe dysplasia.

## 4 DISCUSSION

Clinical data has demonstrated that the 5‐year survival rate of ESCC, while still low, is improving,^5^ and the disease burden is significant in China. Identifying precancerous lesions of ESCC and conducting regular follow‐ups are the keys to detecting cancer early and reducing the incidence and mortality of ESCC. Therefore, exploring the regular patterns of precancerous lesions and understanding the natural progression of esophageal cancer can help in the development of a more scientific approach for management and care of high risk ESCC patients; this could help us achieve our goal of early detection and prevention of ESCC.

This study is a prospective follow‐up study based on a high‐risk population participating in upper gastrointestinal cancer screenings from 2006 to 2016 in Feicheng, an area with a high incidence of ESCC in China. The subjects with SD/CIS are recommended to be treated and followed up with at least once per year if not treated. The results showed that after a median follow‐up of 2.3 years, 62.2% of the subjects showed improvement and 8.2% showed deterioration. Five mD cases, seven MD cases, and five SD/CIS cases advanced to ESCC after a median follow‐up time of 3.5, 2.3 and 2.2 years, with an incidence density of 0.17, 0.79, and 1.77 per 100 person‐years, respectively. The incidence density of ESCC increased with the increase of the grade of precancerous lesions, and the median time of progression to ESCC decreased with the increase of the grade of precancerous lesions.

In this study, the annual transition probability from MD to mD and SD/CIS were 0.194 and 0.038, respectively; for SD/CIS, the annual transition probability to MD and ESCC were 0.111 and 0.015, respectively. An Italian study of secondary endoscopy in 25 patients with precancerous lesions found the annual transition probability of MD and SD/CIS to be lower than that of this study.^18^ The possible reason for this difference may be the small sample size and short interval of follow‐up in the Italian study. This study also found that the annual transition probability of progress was smallest in each precancerous lesion, which is generally consistent with previous research.^19^, ^20^

This study found that 0.97% (17/1753) of the patients with precancerous lesions developed esophageal cancer after a median follow‐up time of 2.3 years. The results of this study were largely consistent with the results of a previous follow‐up study of 21,111 endoscopic screening participants from three areas with high incidence of esophageal cancer in China. They found that 0.68% (143/21,111) of the precancerous lesions developed into esophageal cancer after 8.5 years of follow‐up.^13^ In the previous study, 682 people were followed up for 3.5 and 13.5 years, the cancer rate of esophageal precancerous lesions was 7.6% and 16.7%, respectively,^8^, ^9^ which was much higher than the results of this study and previous studies.^13^ The reason for this result may be that all the subjects in our study were asymptomatic and had not been cytologically diagnosed. Iodine staining under endoscopy and indicative biopsy was used for examination. Only a multi‐point biopsy was performed on the non‐stained areas, and no sampling was taken on the stained areas (normal mucosa). In the former study,^13^ all subjects underwent cytological diagnosis of esophageal squamous cell dysplasia prior to endoscopy and were sampled at a standard site in the middle of the esophagus where no focal lesions were found. Therefore, the results of this study may be more similar to the real situation of precancerous lesions in areas with high incidence of ESCC in China.

In this study, it was found that the incidence density of esophageal cancer increased with the increase of precancerous lesions, which is largely consistent with previous research results.^8^, ^9^, ^13^ Our study also found that the progression time of precancerous lesions to esophageal cancer was shortened with the increase of lesion grade. Five cases of mD before a mean follow‐up of 76.0 months (median follow‐up time was 77 months), five cases of MD mean follow‐up of 57.4 months (median follow‐up time was 63 months), and eight cases of SD/CIS mean follow‐up of 47.0 months (median follow‐up time of 35 months) after development for superficial esophageal cancer, the result was the same with this study, but progress time interval was longer than this research.^21^ The screening which participants began in 2005–2012 and the follow‐up endoscopy was started in 2012–2014 may cause an increase in the follow‐up time interval. In this study, screening and follow‐up procedures were carried out synchronously. For example, if a patient with SD/CIS in 2006 was not treated, he/she would be followed up with in 2007.The median follow‐up time in this study was 2.3 years, and the time interval of progression in this study was closer to the true natural history of esophageal cancer.

GLOBOCAN2018 shows that approximately 70% of esophageal cancer cases occur in men, and the incidence among Chinese men is among the top five in the world.^1^ Previous studies on the risk factors of esophageal cancer have shown that gender and age are closely related to the occurrence of esophageal cancer, males and the elderly at higher risk for esophageal cancer.^22^, ^23^ In previous research, men had 2.4 times higher ESCC incidence than women (0.98 vs. 0.41), and the 50–69 age group had a 3.1 times higher ESCC incidence than that of the 40–49 age group (0.96 vs. 0.31).^13^ In this study, the incidence density of ESCC in males was 2.6 times higher than that of females (0.58 vs. 0.22), and the incidence density of ESCC in the elderly (56–69 age group) was 1.2 times higher than that of the younger population (40–55 age group) (0.45 vs. 0.39), which was consistent with previous research results and reaffirmed that the risk of ESCC in males and elderly people was higher than that of females and younger people.

Previous studies have suggested extending the follow‐up interval of mD and MD with unobvious endoscopy to 5 and 3 years, respectively, differing from this study. The previous study was based on only 4.5% of MD progressing to ESCC after 8.5 years of follow‐up. For SD/CIS, the cumulative 11‐year incidence of the untreated group was 3.3 times higher than that of the treated group. Kaplan–Meier survival analysis showed that endoscopic treatment could significantly improve the survival rate of ESCC in SD/CIS patients, and that timely clinical treatment should be strongly recommended for SD/CIS patients.^24^ Therefore, we suggest that SD/CIS is a necessary endpoint for follow‐up. The results showed that 1.5% of mD progressed to SD/CIS after a median time of 1.4 years, and 8.8% of MD progressed to SD/CIS after a median time of 2.3 years, with an annual transition probability of 0.038. It is reasonable to shorten the follow‐up interval for mD and MD patients to once every 2 years and to conduct clinical treatment for SD/CIS patients.

Strengths of this study include the large population‐based long‐term follow up and the screening is being performed in the general population. Limitations of the study included the 46% compliance of those invited to undergo screening, the small number of patients with SD/CIS, the potential difference of different regions.

## 5 CONCLUSION

In conclusion, the study followed up with 1753 patients and found that more than half of the precancerous lesions regressed. The higher the level of precancerous lesions, the higher the incidence of ESCC. A higher incidence of ESCC was observed among males and individuals of older ages. For mD patients, the follow‐up interval should be shortened to every 2 years when health resources and physical conditions of patients permit, so as to closely monitor the progress status and extend the interval of follow‐up to biennially for patients with MD. For SD/CIS patients, timely clinical treatment should be strongly recommended, and the follow‐up of untreated patients should be carried out in strict accordance with the technical plan.

## AUTHOR CONTRIBUTIONS

Dongqing Gao: Study concept and design, data collection, statistical analysis, writing‐ initial draft. Peipei Lu: Study concept and design, data collection, statistical analysis, writing‐initial draft. Dongqing Gao and Peipei Lu contributed equally to this work. Nan Zhang: Statistical analysis, analysis and interpretation of data, writing–critical revision, acquisition of funding. Li Zhao: Data collection, statistical analysis. Jinhui Liu, Jia Yang, and Jingmin Liu: Data collection, analysis and interpretation of data, statistical analysis. Deli Zhao: Study concept and design and writing‐critical revision. JialinWang: Study supervision, interpretation of data, acquisition of funding, and writing–critical revision.

## FUNDING INFORMATION

This study is supported by the National Natural Science Foundation of China (71904109), the National Science Foundation of Shandong Province (ZR2020KH029), the National Science Foundation of Shandong Province (ZR2019PG0060), and Medical and Health Development Plan of Shandong Province (202012070803).

## CONFLICT OF INTEREST

The authors declared no potential conflicts of interest.

## ETHICS STATEMENT

The studies involving human participants were reviewed and approved by the Institutional Ethical Review Board of Shandong Cancer Hospital and Institute (SDTHEC201909001). The patients/participants provided their written informed consent to participate in this study.

## Data Availability

The data that support the findings of this study are available from the corresponding author upon reasonable request.
